# Obstructive Sleep Apnea as a Risk Factor for Venous Thromboembolism: A Systematic Review

**DOI:** 10.7759/cureus.22729

**Published:** 2022-02-28

**Authors:** Rhea Raj, Akil Paturi, Mohamed A Ahmed, Sneha E Thomas, Vasavi Rakesh Gorantla

**Affiliations:** 1 Anatomical Sciences, St. George's University School of Medicine, St. George's, GRD; 2 Internal Medicine, University of Maryland Medical Center, Baltimore, USA

**Keywords:** inflammation, blood hypercoagulability, intermittent hypoxia, pulmonary embolism, deep vein thrombosis, venous thromboembolism, obstructive sleep apnea

## Abstract

Obstructive sleep apnea (OSA), is a prevalent condition characterized by repeated episodes of pharyngeal airway obstruction resulting in hypopnea and apnea episodes during sleep leading to nightly awakenings. OSA is a major contributor to the healthcare burden worldwide due to its high cardiovascular morbidity and mortality. There is growing evidence to support a pathophysiological link between OSA and venous thromboembolism (VTE). The pro-inflammatory state along with intermittent hypoxia that is invoked in OSA is associated with blood hypercoagulability, venous stasis, and endothelial dysfunction leading to deep vein thrombosis (DVT) and pulmonary embolism (PE). In this systematic review, we aim to analyze and assess the available literature on OSA and VTE (or DVT/PE) to determine whether OSA is an independent risk factor for VTE.

## Introduction and background

Obstructive sleep apnea (OSA) is a highly prevalent form of sleep breathing disorder that affects 9-15% of middle-aged adults worldwide and 24% men and 9% women in the United States (US) [1-5]. It is characterized by recurrent episodes of partial or complete upper airway obstruction leading to intermittent hypoxia, hypercapnia, and sleep fragmentation [3,6]. OSA is a public health concern as it is strongly associated with cardiovascular comorbidities such as coronary artery disease, congestive heart failure, myocardial infarction, sudden cardiac death, and stroke while also affecting the quality of life by causing excessive daytime hypersomnolence and neurocognitive dysfunction [2-4]. Even though OSA is considered to be a highly prevalent condition, approximately 82% of men and 93% of women in the US are undiagnosed [3]. Some of the risk factors for developing OSA include obesity, age, male sex, smoking, narrow airways, and endocrine disorders such as hypothyroidism [3,5]. Obesity is considered the most important risk factor for developing OSA because an increase in adipose tissue within the pharyngeal airways makes them more prone to obstruction and collapse [5]. Interestingly, obesity is also a well-implicated risk factor for venous thromboembolism (VTE) [7]. Deep vein thrombosis (DVT), the most common type of VTE, is a condition in which blood clots form within the deep veins of the body, most commonly the femoral, popliteal, and iliac veins [7,8]. Pulmonary embolism (PE) is considered to be the most serious complication of DVT and is associated with a high mortality rate. It is a result of the clot dislodging from the vein and traveling through the right chambers of the heart to eventually lodge within the pulmonary vasculature [8,9]. 

Although the pathophysiology is complex, Virchow’s Triad, first described in 1856 by Rudolf Virchow can be used to elucidate the relationship between OSA and VTE. The triad details three common factors that contribute to the development of thrombosis: hypercoagulation, venous stasis, and vascular endothelial cell damage [10-14]. Since OSA influences all three factors that contribute to Virchow’s triad, there is growing evidence to support a pathophysiological link between OSA and VTE. The two entities also share some common risk factors such as increasing age, obesity, and sedentary lifestyles [15-17]. Through the analysis of existing literature, this article aims to assess current literature on OSA and VTE (or DVT/PE) and determine whether OSA can be considered an independent risk factor for VTE.

Pathophysiology

Inflammation and Blood Hypercoagulability

Hypercoagulation has been suspected as one of the possible pathophysiological links between OSA and VTE. Hemostatic alterations contribute to the risk of clot formation [18]. The intermittent hypoxic episodes seen in OSA are associated with oxidative stress and increased levels of proinflammatory mediators that ultimately cause alterations to the blood coagulation system [4,19-23]. The relationship between the innate immune system and thrombosis, termed immunothrombosis, has increasingly gained importance in the past decade [24]. The activation of coagulation pathways in response to inflammation is thought to be a protective host response to prevent the dissemination of pathogens in the bloodstream [25]. Sleep deprivation has been shown to induce alterations in monocyte proinflammatory cytokine response by inducing the production of proinflammatory cytokines [26]. Studies have also shown that recurrent upper airway obstruction in OSA results in downstream activation of various inflammatory pathways regulated by the transcription factor nuclear factor kappa B (NF-κB) [27]. NF-κB regulates the expression of genes encoding for cell signaling molecules such as interleukin 1 (IL-1), IL-6, IL-8, tumor necrosis factor (TNF)-α, as well as procoagulant factors such as plasminogen activator inhibitor (PAI)-1 and adhesion molecules such as intercellular adhesion molecule 1 [4,27,28]. Taylor et al. found that human adipocytes exposed to intermittent hypoxia showed an increase in NF-κB mediated production of inflammatory adipokines, thus contributing to the procoagulant milieu observed in OSA [28]. This finding is noteworthy as obesity is a common risk factor for both OSA and VTE. The thrombin-antithrombin (TAT) complex is formed when there is increased blood coagulation, reflecting prothrombotic status [29].

Many studies have shown that the levels of coagulation factors such as TAT complex are higher in OSA patients [30,31]. Other factors shown to be elevated in OSA include fibrinogen, clotting factors VII (FVIIa) and XII (FXIIa), and Von Willebrand factor (VWF), all of which play a role in blood coagulation [29]. This shows that chronic inflammation contributes to one of the key factors in Virchow’s triad, i.e., blood hypercoagulation. 

OSA and Altered Blood Flow

The effect of OSA on blood flow is another possible mediator of the pathophysiological link between OSA and VTE. Numerous studies have established an association between OSA and hematocrit levels [32,33]. Choi et al. found significantly increased hematocrit levels (43.5±3.6%) in 111 subjects that had severe OSA (respiratory disturbance index >30) [34]. This could be because hypoxia induces erythropoietin synthesis, which is a glycoprotein responsible for increasing erythrocyte production [35]. Elevated hematocrit levels increase blood viscosity, thus impeding blood flow and contributing to Virchow’s triad. A rise in hematocrit also causes a preferential axial accumulation of red blood cells (RBCs), causing platelets to adhere to the endothelial lining and result in platelet-endothelium activation, ultimately leads to hemostasis and thrombosis [4,36,37]. The central aggregation of RBCs within vessels along with a reduction in stasis also suppresses the release of nitric oxide (NO), which normally inhibits platelet aggregation and endothelial cell activation [36,37]. Few studies have also looked at viscosity itself and noted that higher plasma viscosity was seen in OSA patients [38-40]. Additionally, blood stasis causes RBCs to stack in a typical “rouleaux'' fashion, which further increases blood viscosity and intravascular resistance, thus promoting thrombi formation in vessels that have low flow rates such as deep veins of the lower extremities [36,41,42]. Hemodynamic alterations that affect the laminar flow and the shear stress within the vessel wall can upregulate genes expressed by endothelial cells, predisposing them to atherogenesis and thrombosis [43]. Willenberg et al. found that obese individuals had significantly lower venous shear stress compared to nonobese controls, indicating that abnormal flow parameters within the venous limb circulation increase the risk for subsequent development of VTE [44]. Such findings regarding hemorheological parameters further support the increased prevalence of VTE in the setting of OSA.

OSA and Vascular Endothelial Dysfunction

The endothelial dysfunction that is seen with intermittent hypoxia and sleep fragmentation is another pathophysiological link between OSA and VTE. Recurrent hypoxic states such as those seen in OSA cause endothelial cells to upregulate the synthesis of tissue factor (TF), a protein that initiates the extrinsic pathway of blood coagulation while suppressing the translation of thrombomodulin, a cofactor required for the activation of protein C in the anticoagulant pathway [4]. The recurrent hypoxia/reoxygenation that is seen in OSA is associated with impaired endothelial function and enhanced inflammatory action [45]. More specifically, it has been shown that endothelial dysfunction in OSA is due to a decrease in the protective function of nitric oxide (NO), which normally aids in controlling blood vessel tone and increasing vessel repair capacity [36,43]. NO, with its anti-inflammatory and vasodilatory properties, also plays an important role in maintaining the integrity of the endothelial vascular barrier by safeguarding it from injury [44,45].

Ip et al. were the first researchers to show that circulating NO levels are reduced in OSA patients and that this finding could be reversed with continuous positive airway pressure (CPAP) therapy [46]. Asymmetric dimethylarginine (ADMA) is a potent endogenous inhibitor of endothelial nitric oxide synthase (eNOS), which affects NO production and subsequently causes endothelial dysfunction [47]. Many studies have also shown that levels of ADMA are elevated in OSA patients, supporting that endothelial dysfunction is a key player in the pathogenesis of OSA [48]. Another mechanism by which endothelial function can be impaired in OSA is by the availability of endothelial progenitor cells (EPCs). EPCs are derived from the bone marrow and function to maintain vascular endothelium integrity by contributing to repair mechanisms. Several studies have been done to investigate the effect of OSA on EPCs, and although the results are varied, most of the studies have shown a reduction in EPCs in patients with OSA [49,50]. Since EPOs are known to play an important role in protecting the endothelium from injury and assisting in repair processes, it is logical to presume that a reduction in circulating EPCs would make the endothelium more prone to injury [47]. Endothelial damage by numerous factors such as chronic inflammation, hypoxia, turbulent blood flow, or inflammatory cytokines can also cause persistent platelet activation and hyperaggregability leading to coagulation [24,51-53]. Platelet adhesion is largely mediated by von Willebrand Factor (vWF), a glycoprotein shown to be elevated in OSA patients [54]. Once activated, platelets adhere to areas of vascular injury, release cytokines and chemokines, and eventually leading to thromboembolic disease [24]. Such findings regarding endothelial dysfunction contribute to the final entity in Virchow’s triad, thus supporting the increased incidence of VTE in patients with OSA.

**Figure 1:**
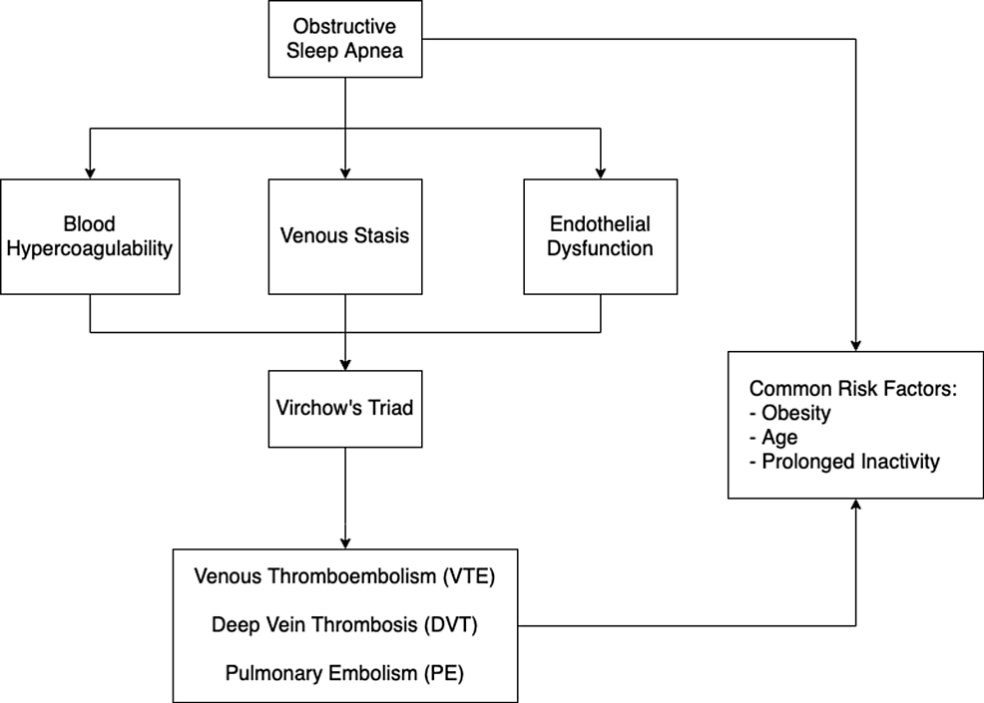
This figure illustrates the pathophysiological link between OSA and VTE. OSA is associated with blood hypercoagulability, venous stasis, and endothelial dysfunction. These three factors contribute to Virchow’s triad and subsequent VTE/DVT/PE formation. The common risk factors between OSA and VTE are also shown. OSA: obstructive sleep apnea; VTE: venous thromboembolism; DVT: deep vein thrombosis; PE: pulmonary embolism.

OSA influences blood coagulability levels, blood flow patterns, and endothelial function - all three of which contribute to Virchow’s triad, thus accentuating the pathophysiology of VTE. Figure 1 illustrates this.

Methods

This systematic review follows the Preferred Reporting Items for Systematic Reviews and Meta-analyses (PRISMA) guidelines [55]. The search for articles was done on February 15, 2022, through four research databases including PubMed, Research4Life, ScienceDirect, and Cumulative Index to Nursing and Allied Health Literature (CINAHL). The following query: "Obstructive sleep apnea" AND "venous thromboembolism" OR "deep vein thrombosis" OR "pulmonary embolism” was used on all search databases. During the screening process, duplicate articles, articles not written in English, and articles published before 2002 were excluded. During the manual screening process, articles were screened based on title, abstract, study type, and full-text availability. It is important to note that some relevant articles might not have been included. Our initial search in the aforementioned databases resulted in 1637 articles. We screened the selected articles according to the inclusion and exclusion criteria and a total of 30 articles were yielded.

Inclusion Criteria

The following inclusion criteria were used: studies written in English and conducted on humans, published from 2002 to 2022, studies relevant to our subject of interest, articles that were peer-reviewed, full texts, and articles including case-control, cohort, and observational studies were included.

Exclusion Criteria

**Figure 2:**
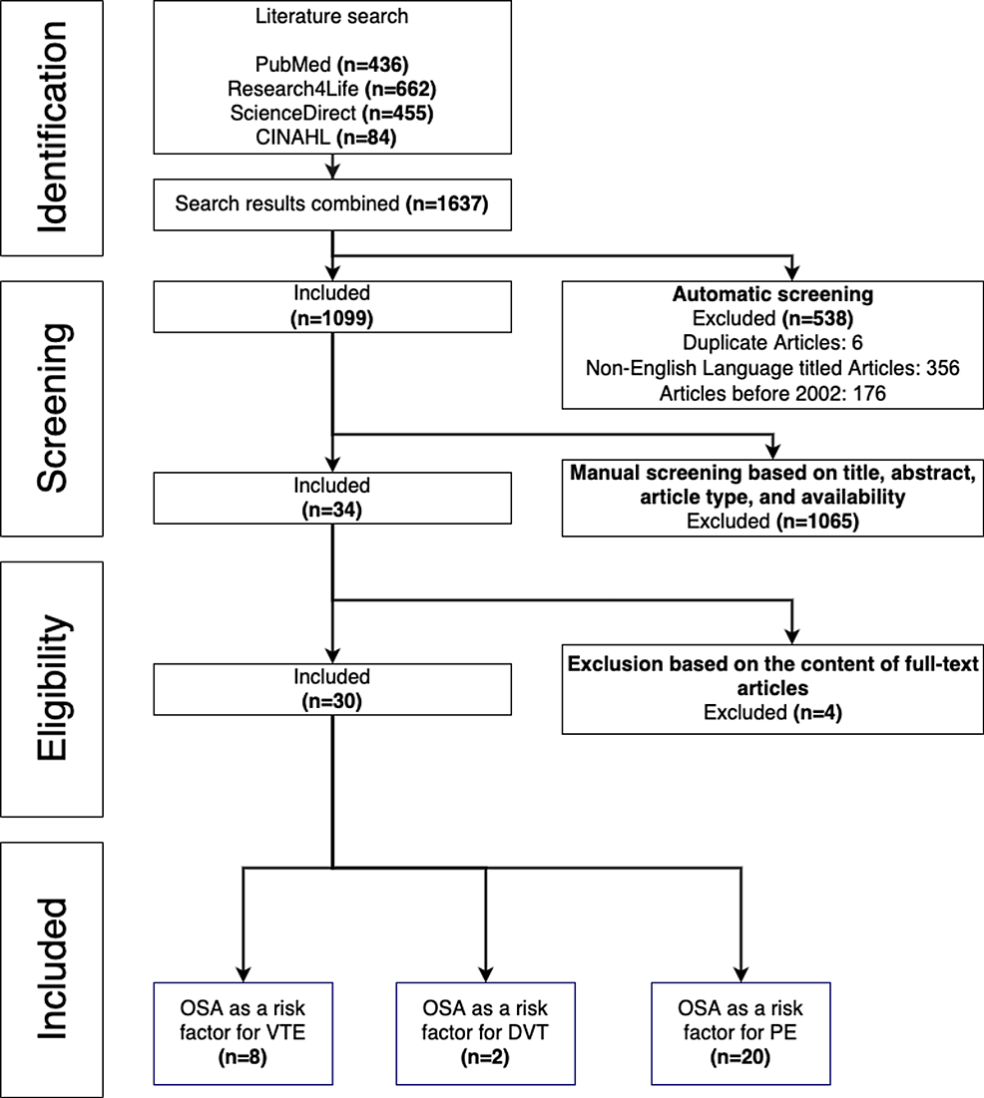
PRISMA flowchart of the systematic review for OSA being an independent risk factor for VTE. OSA: obstructive sleep apnea, VTE: venous thromboembolism, DVT: deep vein thrombosis, PE: pulmonary embolism; PRISMA: Preferred Reporting Items for Systematic Reviews and Meta-Analyses The screening performed for this literature review follows guidelines in the PRISMA statement [55].

Articles that were not primary research studies, articles that were not in English, case reports, review articles, systematic reviews, or articles that were published before 2002 were excluded. All non-full text articles and duplicates were also excluded. The inclusion and exclusion process is illustrated in Figure 2.

Results

In total, 1637 publications were found; 436 from PubMed, 662 from Research4life, 455 from ScienceDirect, and 84 from CINAHL. Among the exclusions were 356 non-English articles that were were found and removed, 176 publications from before 2002 were excluded, and six duplicate articles were removed. This resulted in a total of 538 articles being excluded in the initial automatic screening process, leaving 1099 articles for manual screening. Articles were manually screened on the basis of title, abstract, article type, and availability, leaving 34 articles to be checked for eligibility. Ultimately, 30 articles were included in this review.

## Review

There is growing evidence to suggest that OSA is a risk factor for the development of subsequent VTE (DVT/PE). Studies have suggested that intermittent hypoxia along with the cytokine-signaling cascade observed in OSA induces a prothrombotic state by increasing blood coagulability. Endothelial dysfunction, along with disruptions in venous flow also contribute to the underlying pathophysiology of OSA and VTE.

OSA was found to be a risk factor for the development of VTE (or DVT/PE) in our present review. We looked at 30 peer-reviewed primary research papers and identified a statistically significant link between OSA and subsequent VTE (or DVT/PE). We also discovered evidence to support that blood hypercoagulability plays a major role in the mechanistic pathways that link OSA and VTE, implying that more studies are needed to develop prophylactic therapeutic regimens to minimize adverse PE-related outcomes. A similar review by Alonso Fernández et al. concluded that OSA is highly prevalent in VTE patients and considering it as an independent risk factor would enable clinicians to predict thromboembolic events, thereby preventing adverse outcomes [56].

Chou., et al. found that patients with OSA were 3.113 times more likely to suffer from DVT compared to the pair-matched control group. This study concluded that sleep apnea is an independent risk factor for DVT [57]. Similar results were reported by various studies that supported a significant and independent association between OSA and subsequent PE (DVT/VTE) [58-67]. As this trend was observed in various large-scale studies, there is convincing evidence to support the independent association between OSA and VTE. Conversely, only one study included in this review reported that OSA prevalence was found to be slightly, but not significantly higher in PE patients who underwent knee arthroplasty [68]. In OSA patients requiring CPAP treatment, the incidence of DVT is found to be higher, which suggests that the severity of OSA is associated with a higher risk of DVT [57]. These findings are consistent with those found by Abd El-Azem, as his study reported that 67% of patients who developed VTE were diagnosed with severe OSA (apnea-hypopnea index (AHI)≥30/h) [69]. Similar findings were also reported by Bahar et al., where a 2.3-fold increase in DVT was observed among patients with severe OSA (AHI>30) [70]. This trend was also supported by other studies that concluded that the severity of OSA was associated with the severity of PE [71-73]. Ghiasi et al. found that complications of OSA such as hypertension, rather than OSA itself, increased 30-day mortality in PE patients, suggesting that external factors are associated with the increased mortality in PE patients with OSA [74].

Interestingly, Jiang et al. found that patients with OSA required a higher dose of anticoagulant drugs such as warfarin compared to patients without OSA. They also reported an increased incidence of PE recurrence after warfarin was discontinued, thus supporting that hypercoagulability may be the underlying pathophysiological link between OSA and subsequent PE [4,75]. Similarly, Abd El-Azem found that patients with OSA had higher D-dimer levels, suggesting increased blood coagulability in those patients, as also reported by Bahar et al. [69,70]. Berhgaus et al. and Konnerth et al. think that OSA might be responsible for the hemodynamic alterations observed in PE patients, which provides evidence to support a pathological link between the two conditions [76,77]. Hong et al. found that patients with severe OSA had a shorter prothrombin time (PT) compared to the control group, implying that the severity of OSA is associated with increasing blood coagulability [18]. Blood hypercoagulation increases the likelihood of consequential development of PE, which is the third most frequent cause of cardiovascular disease [78]. Alonso-Fernández et al. believe that the significant and independent association of OSA and subsequent PE represents a major public health issue due to the high mortality rates of both conditions [78]. The intermittent hypoxia observed in OSA might have prothrombotic effects that contribute to the development of VTE, which supports the existence of a hypercoagulable state in OSA patients [79]. Lin et al. and Mao et al. agree that early diagnosis of VTE in OSA patients and prophylactic therapy would help in reducing adverse clinical outcomes [80,81]. More randomized clinical trials on this topic area would be useful to determine anticoagulant prophylactic regimens to reduce adverse PE outcomes in OSA patients.

Obesity is considered the most important risk factor for OSA as the increase in adipose tissue within the pharynx makes it more prone to obstruction, thus causing the clinical manifestations of OSA [5]. Obesity is also an independent risk factor for VTE, as concluded by Hotoleanu, who found a 6.2-fold increased risk in the occurrence of VTE in obese patients [7]. Various studies analyzed in this systematic review reported that the risk of concomitant OSA and VTE occurrence was higher in obese patients such as the retrospective cohort study by Bosanquet et al. [82]. However, Abd El-Azem attributed the increased risk of OSA and subsequent VTE to the underlying pathophysiological link between the two conditions, rather than the effects of obesity alone. He concluded that obesity and OSA worked synergistically to aggravate the risk of VTE [69]. These findings are consistent with other studies which concluded that OSA was an independent risk factor for PE recurrence even after confounding variables such as BMI were adjusted [83,84]. More large-scale studies would be useful to confirm that the relationship between OSA and VTE is a causal one, and not due to the presence of common risk factors such as obesity.

Interestingly, after conducting a gender-stratified multivariate analysis, Dabbagh et al. concluded that OSA is an independent risk factor for VTE among females (odds ratio 2.69) but not among males [85]. This gender bias for concomitant OSA and VTE occurrence was reported in two of the studies included in this systematic review, thus posing the question of whether biological sex plays a role in the underlying pathophysiological mechanisms that interconnect OSA and subsequent VTE. Chung et al. found that women with sleep disorders are at a higher risk of developing subsequent VTE compared to men [86], as also reported by Arzt et al. [87]. More comprehensive studies need to be performed to evaluate whether biological sex is a significant confounding variable for OSA and subsequent VTE occurrence.

Xie et al. reported that patients with overlap syndrome (coexistence of obstructive sleep apnea and chronic obstructive pulmonary disease) had a higher risk of PE compared to patients with OSA alone [88]. This finding was not reported in any other study that was analyzed in this review. Seckin et al. found that patients with OSA who were treated with positive airway pressure (PAP) therapy had a 30% reduction in the relative risk for PE recurrence; however, this result was not statistically significant, possibly due to the small sample size of the subcohort [89]. A higher risk of recurrent VTE was reported in patients who are poorly compliant with CPAP therapy [69]. This implies that when OSA is managed with CPAP therapy, it reduces the intermittent hypoxia that is associated with causing the procoagulant state in OSA, thereby reducing the risk of thrombosis. This finding is valuable as it supports the role of hypercoagulability in the pathogenesis of OSA and subsequent VTE. These findings are summarized in Table 1.

**Table 1:** Articles on VTE (or DVT/PE) following OSA diagnosis obtained from database search. OSA: obstructive sleep apnea; VTE: venous thromboembolism; DVT: deep vein thrombosis; PE: pulmonary embolism; CPAP: continuous positive airway pressure; SBD: sleep breathing disorder; AHI: apnea-hypopnea index; PTE: pulmonary thromboembolism; OSAHS: obstructive sleep apnea-hypopnea syndrome; OSAS: obstructive sleep apnea syndrome; INR: international normalized ratio; PT: prothrombin time; PESI: pulmonary embolism severity index; HR: hazard ratio

	Author	Country	Design & Study Population	Findings	Conclusion
1	Le Mao et al., 2020 [80]	Germany	Prospective cohort study n=(4,153)	5.8% (n=241) of patients with PE had OSA. Patients with concomitant OSA and acute PE had a higher 30-day PE-specific mortality (P< 0.01).	Prophylactic therapeutic regimens must be developed as the presence of concomitant OSA and PE has adverse clinical outcomes.
2	Toledo-Pons et al., 2019 [71]	Spain	Prospective cohort study, n=(120)	OSA patients also had a higher pulmonary embolism severity index (PESI) compared to the AHI ≤ 15/hr cohort (P = 0.007).	PE patients with moderate to severe OSA have worse PE clinical severity. AHI is an independent risk factor for worse PESI outcomes.
3	Berhgaus et al., 2015 [76]	Germany	Prospective cohort study n=(106)	7.5% of patients were diagnosed as having high-risk pulmonary embolism (PE). Frequency of high-risk PE was significantly higher among patients with moderate to severe OSA (P = 0.005).	OSA is a common comorbidity in patients with PE and may contribute as a risk factor for the hemodynamic alterations observed in PE.
4	Jiang et al., 2015 [75]	China	Prospective cohort study n=(97)	Patients with OSAHS require higher doses of warfarin compared to their non-OSAHS counterparts (P < 0.001).	OSAHS patients appear to have a procoagulant state and require a more aggressive anticoagulant treatment regimen to prevent recurrence of PE.
5	Chou et al., 2012 [57]	Taiwan	Non-randomized, pair-matched cohort study, n=(10,185)	Patients with sleep apnea had a 3.113x increase in subsequent DVT (P < 0.002). The incidence of DVT was higher in patients with OSA that required CPAP treatment (P< 0.001).	Sleep apnea was identified as an independent risk factor for subsequent DVT, and patients with severe sleep apnea may be at a higher risk for DVT.
6	Alonso-Fernandez et al., 2013 [58]	USA	Case-control study, n=(209)	The AHI was significantly higher in patients with PE (P< 0.001). 33.6% (n=36) of patients had idiopathic PE.	OSA prevalence is higher in PE patients and there is an independent and significant association between OSA and PE.
7	Konnerth et al., 2018 [77]	Germany	Observational cohort study, n=(253)	Frequency of moderate to severe OSA was higher in high-risk PE patients (P = 0.006). PE patients with moderate to severe OSA had significantly higher D-dimer levels (P = 0.024) compared to patients without OSA.	Acute PE may present more severely when coupled with OSA due to pathophysiological mechanisms such as OSA-related hypoxemia and hypercoagulability.
8	Berhgaus et al., 2016 [79]	Germany	Prospective cohort study, n=(206)	Patients with moderate OSA had a 3.75-fold higher risk of acute PE compared to patients with mild OSA (P < 0.001.). Patients with moderate or severe OSA had significantly lower mean and nadir asleep saturation (P < 0.01 and P < 0.001, respectively).	Likelihood of sleep-related acute PE manifestations is significantly associated with the severity of OSA. Intermittent hypoxia seen in OSA might have prothrombotic effects which lead to VTE.
9	Lin et al., 2013 [81]	Taiwan	Prospective matched-cohort study, n=(15,664)	Risk of developing VTE during the five-year follow-up period was 2.07 times greater in OSA patients than in pair-matched controls after adjusting for confounding variables such as gender, age, and obesity.	Patients with OSA have an increased risk of subsequent DVT in the first five years of their diagnosis, and early recognition and therapy would help in diminishing adverse outcomes.
10	Chung et al., 2015 [86]	Taiwan	Population-based study, n=(139,113)	Incidence of VTE was higher in patients with sleep disorders compared to patients without sleep disorders (adjusted HR of 1.79, 95% CI, 1.49–2.16). Women with sleep disorders are at a higher risk of developing subsequent VTE compared to men (adjusted HR of 2.19, 95% CI, 1.74–2.74).	Patients with sleep disorders are at a higher risk of developing subsequent VTE and sleep-disorder management is important to reduce the incidence of VTE.
11	Alonso-Fernández et al., 2016 [83]	Spain	Prospective cohort study, n=(120)	OSA patients with a previous PE episode had a higher risk of PE recurrence compared to patients without OSA (P = 0.026).	OSA is an independent risk factor for recurrent PE. OSA patients with recurrent PE events should continue anticoagulation treatment as they present with a persistent hypercoagulable state.
12	Bahar et al., 2019 [70]	Turkey	Prospective cohort study, n=(239)	The rate of D-dimer positivity was found to be 17.6% higher in patients with OSAS compared to the control cohort (P = 0.034). The overall prevalence of DVT in OSAS patients was 2.2%.	OSAS is a significant risk factor for subsequent DVT, and patients with severe OSAS should be evaluated for DVT symptoms and possible prophylaxis.
13	Peng et al., 2014 [59]	Taiwan	Retrospective population-based cohort study, n=(38,621)	The risk of DVT was 3.50 fold higher (95% CI = 1.83–6.69) in OSA patients compared to the control cohort. The risk of PE was 3.97 fold higher (95% CI = 1.85–8.51) in OSA patients compared to the control group.	OSA remains an independent risk factor for subsequent DVT and PE after adjusting for age, sex, and other comorbidities.
14	Abd El-Azem, 2019 [69]	Kuwait	Prospective cohort study, n=(107)	A total of 72 patients were diagnosed with OSA and 25% (n=18) of them developed subsequent VTE; 67% (n=12) of the patients who developed VTE had severe OSA (AHI≥30/h).	The occurrence and recurrence of VTE are due to the underlying pathophysiological effects of OSA. The severity of OSA is associated with an increased risk of VTE.
15.	Ghiasi et al., 2015 [74]	Iran	Prospective cohort study, n=(137)	There was no association between OSA and 30-day mortality (P = 0.389) in PE patients. Complications of OSA such as hypertension increased the risk of 30-day mortality among patients with PE (P = 0.003).	Complications of OSA, rather than OSA itself, are associated with an increase in 30-day mortality among patients with PE.
16.	Seckin et al., 2020 [89]	USA	Retrospective cohort study, n=(25,038)	Frequency of acute PE in patients with OSA was 2.4% (P < 0.001). After confounding variables were adjusted, OSA remained an independent risk factor for PE occurrence (P= 0.017).	OSA is an independent risk factor for the occurrence of acute PE, however, OSA does not have a significant effect on hospital mortality among PE patients.
17.	Kezban et al., 2012 [60]	Turkey	Cross-sectional study, n=(30)	Prevalence of OSA in patients with PTE was higher (57%) than in the general population (1-5%). In patients diagnosed with PTE without any known VTE risk factors, OSA was the only significant risk factor (P = 0.045).	PTE patients with OSA symptoms must be evaluated for OSA as there seems to be a significant relationship between OSA and PTE.
18.	Bosanquet et al., 2011 [82]	USA	Retrospective cohort study, n=(840)	Prevalence of OSA in patients with VTE was 15.5% (n=130). Concomitant occurrences of VTE and OSA were associated with obesity (P< 0.001).	There is a link between OSA and venous thrombotic disorders, and obesity is one of the confounding variables.
19.	Hong et al., 2017 [18]	Korea	Retrospective cohort study, n=(146)	Patients with severe OSA had a shorter PT compared to the control group (median difference, 0.59; 95% CI, 0.14 to 1.03).	Patients with moderate to severe OSA have higher blood coagulability markers compared to the general population, suggesting that the severity of OSA is associated with an increased procoagulant state.
20.	Geissenberger et al., 2020 [72]	Germany	Prospective cohort study, n=(101)	All patients enrolled were diagnosed with acute PE (n=101). Patients with moderate to severe OSA had a higher PE severity score (P < 0.001).	OSA is associated with disease severity and survival in patients with acute PE.
21.	Xie et al., 2019 [88]	China	Retrospective cohort study, n=(1,939)	Patients with overlap syndrome (coexistence of obstructive sleep apnea and chronic obstructive pulmonary disease) had significantly higher odds of PE (P < 0.001).	OS is more closely associated with the prevalence of PE than OSA alone.
22.	Epstein et al., 2010 [61]	USA	Prospective cohort study, n=(270)	Patients diagnosed with PE had a higher prevalence of snoring (P = 0.001) and an increased risk of having OSA (P < 0.001) compared to patients without PE.	Findings support that OSA may be an independent risk factor for the development of PE.
23.	Arzt et al., 2012 [87]	Germany	Prospective cohort study, n=(164)	SBDs were found to be more prevalent in patients with DVT and/or PE (P = 0.046). This association was significant in females (P = 0.042), but not in males (P = 0.391).	SBDs are more prevalent in females with DVT and/or PE than in pair-matched controls and are independently associated with thromboembolic events.
24.	Zhang et al., 2012 [84]	China	Retrospective cohort study, n=(58)	PTE patients diagnosed with OSAHS had a higher BMI (P < 0.001), lower age of onset of disease (P < 0.01), and a higher smoking index (P < 0.05).	PTE patients with OSAHS were associated with more severe disease outcomes and should be offered anticoagulant medications and CPAP therapy.
25.	Xu et al., 2020 [73]	China	Meta-analysis, n=(1,570)	PE patients with OSA were more likely to have recurrent PE compared to patients without OSA (RR = 3.87, 95% CI, 1.65-9.07).	Patients with moderate to severe OSA have a significantly increased risk for high-risk PE and recurrent PE and may need more aggressive treatment.
26	Mraovic et al., 2010 [68]	USA	Retrospective, case-control study n=(7,282)	There was no significant association between OSA prevalence in PE patients (6.5% vs 5.4%; P = 0.593).	No significant relationship between the prevalence of OSA in patients with PE undergoing arthroplasty.
27	Sapala et al., 2003 [62]	USA	Retrospective, observational study n=(5,554)	OSA was considered a significant risk factor for the development of postoperative VTE. There was a high prevalence of OSA in patients with PE (33%).	OSA is associated with postoperative VTE.
28	Kosovali et al., 2013 [63]	Turkey	Case-control study n=(73)	The AHI was significantly higher in patients with PE (P =0.010). Severe OSA was found in 21.4% of the PE group but in no controls ( P =0.015).	OSA is highly prevalent and more severe in subjects with PE compared with control subjects.
29	D’Apuzzo et al., 2012 [64]	USA	Case-control study n=(258,455 patients including 16,608 OSA patients)	OSA patients were twice as likely to develop PE when compared to controls(odds ratio, 2.02; 95% CI, 1.3-2.9; P < 0.001). OSA remained an independent risk factor for PE after adjustment for confounding variables (OR, 2.02; 95 % CI, 1.3–2.9).	OSA is an independent risk factor for postoperative PE development.
30	Louis et al., 2014 [65]	USA	Retrospective, cross-sectional study n=(55,781,965)	OSA was significantly associated with pregnancy-related morbidities such as PE (OR, 4.5; 95% CI, 2.3-8.9).	OSA is an independent risk factor for pregnancy-related morbidities including PE.

## Conclusions

In conclusion, compelling evidence exists to support that OSA can be considered an independent risk factor for VTE. Current epidemiological data suggest that the incidence of VTE in the setting of OSA is strikingly high. We thoroughly analyzed 30 peer-reviewed, primary research publications and found that all but one reported a statistically significant increase in VTE (or DVT/PE) incidence in patients diagnosed with OSA. Statistical trends also suggested that patients with severe OSA that required CPAP therapy or additional supportive treatments were at a higher risk for developing VTE, further confirming the importance of OSA being an independent risk factor for VTE. The high prevalence and mortality rates of OSA and VTE make it important for more primary research studies to be carried out to clarify the complex interrelationships of both conditions. Since obesity is a major risk factor for both conditions, further research would be useful to determine that the relationship between OSA and VTE is a causal one, rather than due to shared risk factors. Further comprehensive studies would also prove beneficial in determining prophylactic treatment regimens to minimize the risk of VTE in OSA patients. Lastly, more research would also aid in the global understanding of the underlying pathophysiology that interconnects OSA and VTE.
